# Monitoring Sustainable Development Goals 3: Assessing the Readiness of Low- and Middle-Income Countries

**DOI:** 10.15171/ijhpm.2019.134

**Published:** 2019-12-18

**Authors:** Joy Belinda Nabukalu, James Avoka Asamani, Juliet Nabyonga-Orem

**Affiliations:** ^1^HealthNet Consult, Kampala, Uganda.; ^2^World Health Organization (WHO), Inter-Country Support Team for Eastern & Southern Africa, Harare, Zimbabwe.

**Keywords:** Monitoring, Evaluation, Sustainable Development Goal 3, Universal Health Coverage, Low- and Middle-Income Countries

## Abstract

**Background:** The Millennium Development Goals (MDGs) availed opportunities for scaling up service coverage but called for stringent monitoring and evaluation (M&E) focusing mainly on MDG related programs. The Sustainable Development Goals 3 (SDGs) and the universal health coverage (UHC) agenda present a broader scope and require more sophisticated M&E systems. We assessed the readiness of low- and middle-income countries to monitor SDG 3.

**Methods:** Employing mixed methods, we reviewed health sector M&E plans of 6 countries in the World Health Organization (WHO) Africa Region to assess the challenges to M&E, the indicator selection pattern and the extent of multisectoral collaboration. Qualitative data were analysed using content thematic analysis while quantitative data were analysed using Excel.

**Results:** Challenges to monitoring SDG 3 include weak institutional capacity; fragmentation of M&E functions; inadequate domestic financing; inadequate data availability, dissemination and utilization of M&E products. The total number of indictors in the reviewed plans varied from 38 for Zimbabwe to 235 for Zanzibar. Sixty-nine percent of indicators for the Gambia and 89% for Zanzibar were not classified in any domain in the M&E results chain. Countries lay greater M&E emphasis on service delivery, health systems, maternal and child health as well as communicable diseases with a seeming neglect of the non-communicable diseases (NCDs). Inclusion of SDG 3 indicators only ranged from 48% for Zanzibar to 67% for Kenya. Although monitoring SDG 3 calls for multisectoral collaboration, consideration of the role of other sectors in the M&E plans was either absent or limited to the statistical departments.

**Conclusion:** There are common challenges confronting M&E at county-level. Countries have omitted key indicators for monitoring components of the SDG 3 targets especially those on NCDs and injuries. The role of other sectors in monitoring SDG 3 targets is not adequately reflected. These could be bottlenecks to tracking progress towards SDG 3 if not addressed. Beyond providing compendium of indicators to guide countries, we advocate for a more binding minimum set of indicators for all countries to which they may add depending on their context. Ministries of Health (MoHs) should prioritise M&E as an important pillar for health service planning and implementation and not as an add-on activity.

## Introduction

Health systems performance assessment (HSPA) through monitoring and evaluation (M&E) systems, has been a challenging enterprise which has attracted significant efforts in terms describing the processes, development of frameworks and tools, capacity building as well as recommendations of suitable indicators and information systems among others^1-12^ – a testament of the importance of the subject matter. With ambitious global efforts towards universal health coverage and (UHC) and the Sustainable Development Goals (SDGs), the need for more comprehensive HSPA through effective M&E has become even greater.^13^ HSPA is a process that seeks to undertake a “health check” of the entire health system using statistical indicators, and links health outcomes to the strategies and functions of the health system.^11^ Monitoring is here defined as routine tracking and reporting of priority information about a program and its intended outputs and outcomes^12^ while evaluation measures how well the program has met expected objectives.^14^ The assessment of how well a health system has achieving desired results, which in this manuscript we refer to as HSPA, is assessed through M&E. Increasingly, it is recognised that comprehensive and transparent M&E is required not merely as a demonstration of accountability and evidence of the effectiveness of interventions, but also to make the case for additional health sector resources.^12^ Therefore, comprehensive M&E has become an integral element of every health sector strategic plan and much so for the UHC and SDGs agenda.

Experiences from the Millennium Development Goals (MDGs) showed that regular monitoring was emphasised on MDG related programs and targets (maternal and child health, HIV, tuberculosis and malaria) and more attention paid to global reporting without much integration with the overall health sector M&E needs of the country. This resulted in a multiplicity of monitoring indicators and vertical information systems to satisfy specific reporting obligations and in some cases meet the requirements of funders.^15^ The new direction of the global health agenda towards SDGs and UHC which embraces the broader socio-economic determinants of health and equity considerations presents broader and more sophisticated monitoring requirements. In this respect, the role of other sectors in the attainment of health-related targets and for that matter their incorporation in health sector performance assessments has become much more paramount. Embracing information sources from both within and without the health sector which is a characteristic of this multi-sectoral and inclusive approach advocated for by the SDGs is crucial. In this context, several critical components are relevant to HSPA including the activities involved – defined as all activities whose primary purpose is to promote, restore and/or maintain health irrespective of who undertakes these; and the people, institutions and resources involved in undertaking the activities, as well as how they are arranged and managed.^10^ This would concomitantly come with its own challenges or at least accentuate lingering M&E challenges within countries.

Previous works drawing from international experiences have well-documented a myriad of challenges affecting comprehensive M&E leading to weak HSPAs.^15-18^ Some of these include the lack of good quality data, data incompleteness, poor timeliness to inform decision-making and exclusion of data from the private sector among others.^17^ On the other hand, high political interest and stewardship, clarity of vision for M&E with roles and responsibilities of actors spelt out, the involvement of stakeholders and strengthening of existing information systems as opposed to creating new ones are some of the promising lessons from countries.^18,19^ Nevertheless, as part of efforts to track the health-related SDGs and UHC, countries are expected to develop M&E plans to track implementation of, and attainment of targets in their health sector plans. A review and synthesis of the challenges to be faced by countries in the SDG era would be useful for planning cross-country interventions for strengthening M&E. There has however been limited documentation or synthesis across countries to understand their own assessment of M&E challenges and priorities especially in the context of Africa.

Towards a harmonised and comprehensive HSPAs, the International Health Partnership (IHP) (which evolved into UHC2030) developed a guide to be used by countries to develop their health sector M&E plans as a component of the health sector strategic plan, covering disease programs and health system actions.^10^ As such, a comprehensive HSPA is embedded primarily in a comprehensive M&E plan. Therefore, the IHP technical guidance urges countries to develop M&E plans that adopt a systematic results-chain representation comprising of four major domain/categorisation indicators namely, system inputs and processes, outputs, outcomes, and impact covering disease programs and health system strengthening interventions (eg, recruitment of health workers, putting information systems in place).^10^ Despite the international guidelines and other frameworks,^7,9,15,16,18^ the prioritisation of which indicators to select for the monitoring of health and health-related interventions is almost entirely the prerogative of countries. It is of interest however, to examine the pattern of country-level indicator selection especially in the light of the SDG 3 targets, an enterprise that has received limited intellectual effort.

As part of efforts to expand the body of knowledge and strengthen efforts for HSPA through effective M&E, we analysed the health sector M&E plans of 6 African countries to synthesise their challenges, priority indicators selected for monitoring SDG 3 and UHC and consideration of other sectors in M&E. It is worth emphasising that the intent was not to perform a quality appraisal of the M&E plans using a ‘set of standards’ but to provide a synthesis of common challenges confronting M&E in countries and explore their priorities based on the pattern of indicator selection amongst the countries. We acknowledge that some of the SDG 3 indicators may be monitored through other M&E frameworks, but we restrict our review to health sector M&E plans since our focus is on SDG 3 and, further note that the health sector goes beyond Ministry of health (MoH).

## Methods

A review of M&E plans of 6 countries within the World Health Organization (WHO) Africa Region (see Table 1) was conducted to synthesise the challenges and to identify indicator selection patterns. Here pattern refers to how many indicators were selected, selection of indicators for monitoring disease programs and health systems strengthening, and the selection of indicators for the different domains in the results chain. Consideration for the results chain derives from the need to show how inputs into the system (eg, financing, infrastructure) and processes (eg, supply chain) result into outputs (such as availability of services and interventions) and eventual outcomes (eg, intervention coverage) and impact (eg, improved health outcomes).^12^ The countries were conveniently selected if they had current and publicly available health sector strategic plan to which the M&E plans is aligned, M&E plan was also publicly available, the M&E plan covered the post-MDG period (after 2015), and country belonged to the WHO African Region. Based on these criteria, 6 countries were selected for review and analysis. The review of each M&E plan was conducted independently by two persons guided by the format provided by the WHO/IHP+.^12^ We kept the classification of indicators as initially classified the by countries in the different domains of the results chain. Indicators were further broadly grouped by countries into health systems, service delivery, communicable diseases, non-communicable diseases (NCDs) and those that measure overall population health status including maternal and child health among others.

**Table 1:** Country M&E Plans Reviewed

**Country**	**Title of M&E Plan**	**Timeframe**
Guinea	PNDS evaluation du	2015-2024
Kenya	Health sector M&E framework	2014-2018
The Gambia	The national M&E plan for the National Health Strategic Plan	2014-2020
Uganda	M&E plan for implementation of the health sector development plan	2015/2016-2019/2020
Zanzibar	M&E framework for health sector strategic plan III	2013/2014-2018/2019
Zimbabwe	Zimbabwe health sector performance M&E policy guidelines and strategy	2016-2020

Abbreviations: M&E, monitoring and evaluation; PNDS, Plan national de suivi.

### Data Analysis

Two levels of analysis were conducted in line with the objective of the study. First, we manually conducted a content analysis of the situation analyses of the selected M&E plans to thematically synthesise the main challenges confronting M&E within the countries. The M&E plans were read by two people at different times in which each reviewer coded similar descriptions, phrases and words manually. The coded texts were then extracted into a separate sheet and checked for relevance to the objectives of the paper then mapped into themes. Each theme and subtheme were therefore supported with verbatim quote(s) taken from at least two of the M&E plans reviewed. Finally, the last author reviewed the M&E plans alongside with the identified themes as a confirmability check.

We reviewed the indicators in line with the WHO/IHP+ guidance^12^ which emphasises consideration for:

1. Detailing a logical and results-chain – selection of indicators in the 4 major domain indicators namely, system inputs and processes, outputs, outcomes, and impact. We did not assess the reliability of the indicator selected;
2. Indicators covering diseases programs and health systems strengthening;
3. Inclusion of data sources and frequency of measurement; As well as the WHO guidance^20^ which emphasises:
4. Inclusion of SDG 3 related targets. As part of global efforts to monitor the 13 targets of SDG 3, there are 27 indicators monitoring various aspects of the targets.

Secondly, a descriptive analysis of the indicators selected by countries was conducted to explore the pattern of selection. Subsequently, a parametric analysis using zero-order Pearson correlation was conducted using Statistical Package for Social Science (SPSS) to examine if the selection of a particular type of indicator was associated with the inclusion or exclusion of other types of indicators. Association between the different variables were considered statistically significant at 0.05 criterion level.

### Data Availability

All the reviewed M&E plans are in the public domain.

### Patient and Public Involvement

Our study reviewed documents that were in the public domain. There was no involvement of patients or the public.

## Results

The results of the review are presented in two main sections. The first section provides a synthesis of the challenges confronting M&E as identified in the situation analyses of reviewed plans. The second section is concerned with the analysis of the number and pattern of M&E indicators that were selected by countries including an assessment of the number of indicators from each country that are aligned with the SDG 3 monitoring indicators.

### Thematic Synthesis of Monitoring and Evaluation Challenges Within Countries

Content analysis of the situation analysis components of the M&E plans revealed common themes of challenges across the countries. These include weak institutional capacity and organisational structures for M&E in countries; inadequate coordination of partners; insufficient funding for M&E; inadequate dissemination and use of M&E products for decision-making.

#### Weak Institutional Capacity and Organisational Structures for Monitoring and Evaluation

The situation analyses of the M&E plans revealed pervasive weaknesses in the institutional capacities of Ministries of Health (MoHs) for M&E owing to inadequate skilled personnel in M&E and suboptimal attention being paid to M&E functions when defining the structure and organograms at the MoHs. These gaps tend to be exacerbated at the subnational levels where in some cases, there are no designated officers to undertake M&E functions. For example, in Gambia’s M&E plan, it was bemoaned that “… there are inadequate resources (human, material and financial) to support M&E [resulting in] no reporting from the teaching hospital and the general hospitals unless the central team goes to actively collect data, which is often late…”^21^ Whereas in Uganda significant “progress [was made] towards aligning the previously fragmented information systems within the health sector to ensure contribution to the one M&E system” which resulted in improved data completeness and quality,^22^ however “… the M&E Structure for the MoH is not defined to streamline the M&E functions [particularly the] … absence of a human resource compliment for M&E at the MoH … [which] there is still [the] need for skills development for M&E” (p. 14). Similarly, in the case of Zimbabwe, “the human resource structures at provinces, districts and hospitals, respectively, are not formally structured to include the Provincial Health information officers, M&E officers, the District Health information officers and the Hospital Health information officers.”^23^ The foregoing situation was also aptly highlighted in Zanzibar’s M&E plan which noted that “human resources for [M&E] implementation remains inadequate at all levels of the health structure” (p. 15). Also, the penchant of creating parallel project management units or project implementation units within MoHs have also been implicated in weakening the capacity of the MoH’s planning, M&Eunits.^21,22^

#### Fragmentation of Monitoring and Evaluation Function With Inadequate Coordination Mechanisms

Another challenge that cut across the M&E situation analyses of 6 different countries related to the weak coordination of the M&E processes especially within the MoH where roles and functional relationships between the various departments such as information technology, health information/statistics, disease programs and planning departments were not clearly defined in terms of their role in M&E. Even so, external coordination and stewardship with development partners and counterpart Government’s Ministries, Departments and Agencies (MDAs) leaves much to be desired. In the Gambia for instance, it was pointed out that “the roles, responsibilities are not very clear …with minimal coordination between the different technical units, the directorates, the broader health sector stakeholders and the sub-national levels, especially the regions”^21^ similar to Guinea^24^ where “there are several fragmented health information subsystems … that are managed by different ministries and national institutions with an obvious weakness of coordination” (p. 22). The situation analysis of the Kenyan M&E plan also described the prevailing M&E ecosystem prior to the development of the plan as “… disjointed, with no coordination structures or framework … [where the] numerous programme specific/disease-based M&E systems operate separately, not sharing data and information with each other.”^25^ This “prevailing absence of a unified approach to monitoring programmatic and sector performance has created duplication of effort, inefficiencies, lagging capacity in the analysis of health system performance and in implementing comprehensive M&E” (p. 14). Similarly, it was identified in Zimbabwe that “fragmented/vertical programme specific M&E units are working in silos” (p. 38). These weak linkages are said to have led to “inadequate sharing of information” for health system performance assessment in Zanzibar^26^ and “poor timing in the provision of results for guiding sector planning” in Uganda.^22^ Additionally, low reporting rates from the private sector have been attributed to the weak coordination which in turn limited the ability to appropriately monitor overall sector performance in some of the countries.^21,23,25^

#### Inadequate Domestic Monitoring and Evaluation Financing Resulting in Donor Dependence

Inadequate domestic financing for M&E has created major gaps that impede effective implementation and monitoring of the National Health Strategic Plans.^21^ Undoubtedly, most African countries are faced with insufficient domestic resources to meet all the needed investments in the health sector^27^ resulting in gaps in critical areas including M&E such that even “quarterly reports and reviews between the [MoH and sub-national levels] are mostly donor-funded.”^23^ In Zanzibar, it was re-echoed that “insufficient funds … was the major constraint to [M&E] implementation,” noting that “… the markedly limited government budget for HMIS [Health Management Information System] led to over-reliance on donor project resources often associated with piece-meal initiatives” (p. 14). Even in the case of Uganda where M&E appears to be one of the strong points of the health system, it was noted there are still “irregular program and sub-national performance reviews due to inadequate funds” (p. 15) resulting in “evaluations for most programs not conducted … and as a result impact of interventions [also] not well-documented” (p. 15).

#### Inadequate Data Availability

The unavailability of data was identified as one of the major setbacks of effective M&E across the plans of the countries under review. This has partly been linked to M&E processes intricately anchored on the functionality and quality of HMIS with the aim of ensuring timeliness, completeness and accessibility to all stakeholders. However, the review shows that monthly reporting is not always complete and timely^21,25^ which is sometimes attributed to the inadequate supply of relevant tools for data collection and reporting. Other challenges relate to the misclassification and ultimately inaccurate estimates of disease burden due to limited use of the International Classification of Diseases (ICD). For example in Uganda, only 13 out of 335 health facilities were using the ICD 10. Similarly, in the Gambia, it was identified that “the ICD is not used despite previous recommendations” which coupled with the absence of community-based information system is undermining data quality and hence effective M&E.

#### Inadequate Dissemination and Utilization of Monitoring and Evaluation Products

Another common theme that run across the M&E plans is the phenomenon of minimal use of evidence from routine M&E processes for decision-making. The Zanzibar’s M&E plan, for instance, noted that “… analysis, synthesis, effective dissemination and use of information to guide policy dialogue and implementation of health programmes remain a challenge,”^26^ an assertion that is corroborated by Uganda’s M&E plan which reiterated that “the utilization of data for decision-making is still minimal” (p. 15). The challenge has not only been partly attributed to untimely reporting as noted in the Zimbabwe M&E plan but also an endearing “weak culture of data demand and use of information for decision-making.”^25^

### Descriptive Analysis of Indicators Selected by Countries

The countries reviewed largely followed a systematic results chain framework as proposed by IHP guidance^12,20^ in developing their respective M&E plans by selecting indicators to monitor the use of inputs, processes, outputs, outcomes and demonstrable impact at the population level. In total, 614 indicators were selected by the 6 countries which varied widely from 38 indicators in the case of Zimbabwe to as many as 235 for Zanzibar. As shown in Table 2, across the 6 countries, 55.5% (n = 341) of the selected indicators were not classified by the countries as to whether they were intended to measure inputs/processes, outputs, outcomes or impact, in that, as high as 210 indicators (89%) for Zanzibar and 93 (69%) for The Gambia were not classified. Overall, about 10% (n = 62) of the selected indicators were classified by the respective countries as measuring inputs and processes while 9% (n = 60) were intended to measure outputs; 16% (n = 97) to measure outcomes and 9% (n = 54) to measure impact in terms of population health status. For three of the countries (Uganda, Kenya, and Guinea) that classified all their selected indicators, an average of 22% of the indicators were intended to measure inputs/processes, 21% intended to measure outputs, 39% to measure intermediate outcomes and 18% intended to measure the impact of various interventions on population health status.

**Table 2:** Analysis of the Selection of Core Indicators in Country M&E Plans

**Country**	**Indicator Domain**	**Classification and Number (%) of Indicators**						**Mechanism for Tracking**					
		**Unclassified**	**Input/Process**	**Output**	**Outcome**	**Impact**	**Total Domain**	**Data Sources Indicated**	**The Frequency of Reporting Indicated**	**Baseline** **Provided**	**Equity Stratifies** **Identified**	**Targets Set**
Uganda	Health systems		7 (100)				7 (17)	Y	Y	Y	Y	Y
	Service delivery			13 (50)	13 (50)		26 (62)	Y	Y	Y	Y	Y
	NCDs risk factors				4 (100)		4 (10)	Y	Y	Y	Y	Y
	Health status					5 (100)	5 (12)	Y	Y	Y	Y	Y
	Total		7 (17)	13 (31)	17 (40)	5 (12)	42 (100)					
Kenya	Health systems		32 (80)		8 (20)		40 (40)	Y	Y	Y	Y	Y
	Service delivery		5 (12)	10 (24)	27 (64)		42 (42)	Y	Y	Y	Y	Y
	NCDs risk factors				6 (100)		6 (6)	Y	Y	Y	Y	Y
	Health status					12 (100)	12 (12)	Y	Y	Y	Y	Y
	Total		37 (37)	10 (10)	41 (41)	12 (12)	100 (100)					
The Gambia	Health systems	54 (83)	6 (9)	5 (8)			65 (48)	Y	Y	N	N	N
	Service delivery	39 (64)		13 (21)	1 (2)	8 (13)	61 (45)	Y	Y	For 70 of indicators	N	For 50 of indicators
	NCDs risk factors				5 (100)		5 (4)	Y	Y	N	N	N
	Health status					4 (100)	4 (3)	Y	Y	Y	N	Y
	Total	93 (69)	6 (4)	18 (13)	6 (4)	12 (9)	135 (100)					
Zanzibar	Health systems		4 (57)	2 (29)		1 (14)	7 (3)	Y	Y	Y	Y	N
	Service delivery			3 (30)	7 (70)		10 (4)	Y	Y	Y	Y	Y
	NCDs risk factors				4 (100)		4 (2)	Y	Y	Y	Y	Y
	Health status					4 (100)	4 (2)	Y	Y	Y	Y	Y
	Not classified into domains	210 (100)					210 (89)	N	N	N	Y	N
	Total	210 (56)	10 (3)	23 (6)	17(5)	21 (6)	374 (100)					
Zimbabwe	Health systems	12 (100)					12 (32)	Yes, but not by indicators	Yes, but not by indicators	Y	Geographical and gender	Y
	Service delivery	6 (100)					6 (16)	Not explicitly	Not explicitly	Y	Geographical and gender	Y
	Communicable diseases	8 (100)					8 (21)	Not explicitly	Not explicitly	Y	Geographical and gender	Y
	NCDs	4 (100)					4 (11)	Not explicitly	Not explicitly	N	Geographical and gender	Y
	Maternal and child health	4 (100)					4 (11)	Not explicitly	Not explicitly	Y	Geographical and gender	Y
	Emergencies	4 (100)					4 (11)	Not explicitly	Not explicitly	Y	Geographical and gender	Y
	Total	38 (100)					38 (100)					
Guinea	Health systems		8 (67)	1 (8)		3 (25)	12 (19)	Y	Y	70% of indicators	Y	70% of indicators
	Service delivery			13 (42)	18 (58)		31 (48)	Y	Y		Y	
	Communicable diseases					9 (100)	9 (14)	Y	Y		Y	
	NCDs				4 (80)	1 (20)	5 (8)	Y	Y		Y	
	Health status					7 (100)	7 (11)	Y	Y	Y	Y	
Total	Total		8 (13)	14 (22)	22 (34)	20 (31)	64 (100)					
Overall		341 (56)	62 (10)	60 (10)	97 (16)	54 (9)	614 (100)					

Abbreviations: M&E, monitoring and evaluation; NCDs, non-communicable diseases.

Also, the indicators in the reviewed M&E plans were broadly grouped into health systems, service delivery, communicable diseases, NCDs and those that measure overall population health status including maternal and child health among others. As shown in Table 2, except for Zimbabwe (15.8%) and Zanzibar (4.3%), about 49.4% of the selected indicators of the other four countries were focused on service delivery which ranged from 42% for Kenya to 61.9% for Uganda. Furthermore, between 17% (Uganda) and 48% (The Gambia) of the indicators also focused on health system issues. Only Uganda prioritised nearly 10% of the selected indicators to monitor risk factors for NCDs as the other five countries selected between 4% and 6% of the indicators intended for monitoring NCDs risk factors. Similarly, health status indicators which measure more of the impact of interventions on the population health represented 6% of the total number of indicators, ranging from 4% in three countries (Zimbabwe, Zanzibar, and the Gambia) to 12% in Kenya.

Across the countries, most indicators had baseline values, targets and frequency of reporting but there were also some omissions. For example, The Gambia had some indicators whose baseline were not available, but future targets were set. In the case of Zimbabwe, the source(s) of data for each indicator was not stated explicitly but rather generic data sources were listed including their frequency of data generation/compilation.

In giving effect to the concept of leaving no one behind embedded in the UHC agenda, several equity dimensions in terms of monitoring access to needed services by population sub-classes such as socio-economic status, residence, gender, religion and race were looked out for in the M&E plans reviewed. As shown in Table 2, all the M&E plans made provision for including some equity stratification except for The Gambia’s M&E plan which made no consideration for equity analysis, while Zimbabwe considered only geographical and gender stratification. Surprisingly, however, none of the M&E plans paid attention to monitoring access to services by the elderly.

### Correlation Between Selection of Indicator Types

A linear correlation was conducted to explore the relationships between the pattern of selection of input/process, output, outcome and impact indicators by countries. As shown in Table 3, the results revealed that the countries that had a high number of unclassified indicators in the M&E plan were associated with a significantly decreased number of input/process indicators (r = -0.97); output indicators (r = -0.38) and impact indicators (r = -0.61) but associated with a higher number of outcome indicators (r = 0.98). In contrast, a positive correlation was observed between the selection of inputs/processes indicators on one hand and on the other hand, output indicators (r = 0.12), outcome indicators (r = 0.40) as well as impact indicators (r = 0.31). It is also worth noting that a negative but statistically insignificant relationship (r = -0.04) was observed between the selection of output indicators and that of outcome indicators.

**Table 3:** Correlation Between Types of Indicators Selected by Countries

**Variables**	**Input/Process Indicators**	**Output Indicators**	**Outcome Indicators**	**Impact Indicators**
Unclassified indicators	-0.97^*^	-0.38^*^	0.98^*^	-0.61^*^
Input/process indicators		0.12^*^	0.40^*^	0.31^*^
Output indicators			-0.04^NS^	0.70^*^
Outcome indicators				0.46^*^

*P  < .05; NS, not statistically significant.

It can be inferred from this finding that if the countries were to classify all the remaining unclassified indicators, most of these could have been input/process and impact indicators and a relatively small number being output indicator.

### Alignment of the Country Selected Indicators With Sustainable Development Goal 3 Monitoring Indicators

For country M&E plans under review, we examined the extent of alignment of the country selected indicators with those of the SDG 3 targets. Alignment here means that the country selected that particular indicator but with a country specific target. On average, the M&E plans included 58% of the SDG 3 indicators. Kenya’s M&E plan contained the most (66.7%, n = 18) indicators aligned with SDG 3 followed by Zimbabwe (63%, n = 17) with the least number of SDG 3 indicators being included in the Zanzibar M&E plan (48.1%, n = 13). It is important to point out that none of the M&E plans included any of the SDG 3 indicators that monitors target 3.9 to by 2030, substantially reduce the number of deaths and illnesses from hazardous chemicals and air, water and soil pollution and contamination. Similarly, none of the M&E plans included the only indicator that monitors target 3.a which seeks to strengthen the implementation of the WHO Framework Convention on Tobacco Control in all countries. Only Kenya’s M&E plan included indicators aligned with the monitoring of SDG 3 targets 3.5 and 3.6 which, respectively, seek to strengthen the prevention and treatment of substance abuse, including narcotic drug abuse and harmful use of alcohol; and by 2020, halve the number of global deaths and injuries from road traffic accidents. The foregoing reinforces the finding that countries somehow did not adequately prioritise indicators that support the monitoring of risk factors for NCDs. Nevertheless, the countries included all the indicators meant for tracking targets 3.1 and 3.2 which are both maternal and child health-related targets. See Table 4 for details on the extent of alignment between the country M&E plans and the SDG 3 indicators.

**Table 4:** Analysis of SDG 3 Target Indicators Selected by Countries

**SDG 3 Targets**	**Number of Indicators for SDG Targets**	**Number Selected by Countries**					
		**Uganda**	**Kenya**	**The Gambia**	**Zanzibar**	**Zimbabwe**	**Guinea**
3.1 By 2030, reduce the global maternal mortality ratio to less than 70 per 100 000 live births	2	2	2	2	2	2	2
3.2 By 2030, end preventable deaths of newborns and children under 5 years of age, with all countries aiming to reduce neonatal mortality to at least as low as 12 per 1000 live births and under-5 mortality to at least as low as 25 per 1000 live births	2	2	2	2	2	2	2
3.3 By 2030, end the epidemics of AIDS, tuberculosis, malaria and neglected tropical diseases and combat hepatitis, water-borne diseases and other communicable diseases	5	3	4	4	2	4	4
3.4 By 2030, reduce by one-third premature mortality from NCDs through prevention and treatment and promote mental health and well-being	2	0	1	0	0	1	1
3.5 Strengthen the prevention and treatment of substance abuse, including narcotic drug abuse and harmful use of alcohol	1	0	1	0	0	0	0
3.6 By 2020, halve the number of global deaths and injuries from road traffic accidents	2	0	1	0	0	0	0
3.7 By 2030, ensure universal access to sexual and reproductive healthcare services, including for family planning, information and education, and the integration of reproductive health into national strategies and programmes	2	2	1	2	1	2	1
3.8 Achieve UHC, including financial risk protection, access to quality essential healthcare services and access to safe, effective, quality and affordable essential medicines and vaccines for all	2	2	2	2	2	2	1
3.9 By 2030, substantially reduce the number of deaths and illnesses from hazardous chemicals and air, water and soil pollution and contamination	3	0	0	0	0	0	0
3.a Strengthen the implementation of the WHO Framework Convention on Tobacco Control in all countries, as appropriate	1	0	0	0	0	0	0
3.b Support the research and development of vaccines and medicines for the communicable and NCDs that primarily affect developing countries, provide access to affordable essential medicines and vaccines, in accordance with the Doha Declaration on the TRIPS Agreement and Public Health, which affirms the right of developing countries to use to the full the provisions in the Agreement on Trade-Related Aspects of Intellectual Property Rights regarding flexibilities to protect public health, and, in particular, provide access to medicines for all	3	2	2	2	2	2	2
3.c Substantially increase health financing and the recruitment, development, training and retention of the health workforce in developing countries, especially in the least developed countries and small island developing States	1	1	1	1	1	1	1
3.d Strengthen the capacity of all countries, in particular developing countries, for early warning, risk reduction and management of national and global health risks	1	1	1	1	1	1	1
**Total**	**27**	**15**	**18**	**16**	**13**	**17**	**15**
**Percentage of indicators selected by countries**		**55.6%**	**66.7%**	**59.3%**	**48.1%**	**63.0%**	**55.6%**

Abbreviations: NCDs, non-communicable diseases; UHC, universal health coverage; SDG, Sustainable Development Goal; WHO, World Health Organization; TRIPS, Trade-Related Aspects of Intellectual Property Rights.

### Consideration of Other Sector’s Roles

The health sector is broader than the MoH and indeed the broad SDG 3 agenda and attainment of UHC calls for multi sector collaboration. This implies, health-related activities that are core to the attainment of UHC will be implemented by other sectors and likewise some of the health-related indicators (eg, Death rate due to road traffic injuries) will be housed in other sectors. As shown in Table 5, consideration for multi sectoral collaboration in M&E is limited. Only the statistics unit/department is reflected in 3 of the countries. Consideration for cabinet in The Gambia and Zanzibar is commended given their role in resource allocation.

**Table 5:** Role of Other Sectors in M&E as Reflected in the Plans

**Country**	**Line ministries**	**Roles**
**Uganda**	Ministry of foreign affairs (National identification registration authority)	Operating and managing the Civil Events Registry.
		Timely processing and dissemination of Vital Statistics.
		Capacity building of health providers and community in registration of births and deaths.
		Provision of registration equipment and materials.
	Ministry of Finance – UBoS	Coordinating, supporting, validating and designating as official any statistics produced by UBoS, MDAs, and LGs.
		Coordinating and clearing all censuses and nationally representative household economic surveys.
		Ensuring production, harmonization and dissemination of statistical information.
		Strengthening statistical capacity of planning units in MoH and local governments for data production and use.
		Ensuring best practice and adherence to standards, classifications, and procedures for statistical collection, analysis and dissemination in MoH and LGs.
		Ensuring that complete and approved health statistical data are made easily available to the public in a timely manner, while ensuring that the sharing of reports respects the Access to Information Act, 2005.
**Kenya**	Ministry of Devolution & Planning	Ensure functional linkage with MoH.
**The Gambia**	Cabinet/Parliament	Overall political and policy oversight.
		Review of sector progress in the past year (based on the AHSPR), against the policy imperatives set out in contribution towards the second NHP and NDP.
		The health sector shall interface with parliament and cabinet whenever necessary and during the JRM of the health sector.
	Bureau of Statistics (GBoS)	Coordinating, supporting, validating, and designating as official any statistics produced by GBoS.
		Coordinating and clearing all censuses and nationally representative household economic surveys.
		Ensuring production, harmonization and dissemination of statistical information.
		Strengthening statistical capacity of planning units in MoH and LGs for data production and use.
		Ensuring best practice and adherence to standards, classifications, and procedures for statistical collection, analysis and dissemination in MoH and LGs.
		Ensuring that complete and approved M&E reports and health statistical data are made easily available to the public in a timely manner.
**Zanzibar**	House of Representative/Cabinet/Parliament	Overall political, and policy oversight.
		Review of sector progress in the past year (based on the AHSPR), against the policy imperatives set out in contribution towards MoH achievements.
	The OAG	Carrying out audits and providing reports on public accounts of all public offices and any public corporation or other bodies established by an Act of Parliament.
		Conducting financial, value for money and other audits, such as gender and environment audits, in respect of any project or activity involving public funds.
	Office of Government Statistician Zanzibar	Coordinating, supporting, validating and designating as official any statistics produced by OCGS MDAs and LGAs.
		Coordinating and clearing all censuses and nationally representative household economic surveys.
		Ensuring production, harmonization and dissemination of statistical information.
		Strengthening statistical capacity of planning units in MoH and LGAs for data production and use.
		Ensuring best practice and adherence to standards, classifications, and procedures for statistical collection, analysis and dissemination in MoH and LGAs.
		Ensuring that complete and approved M&E reports and health statistical data are made easily available to the public in a timely manner, while ensuring that the sharing of reports respects the access to information deliberations.
**Zimbabwe**	None stated	
**Guinea**	None stated	

Abbreviations: MoH, Ministry of Health; M&E, monitoring and evaluation; OAG, Office of the Auditor General; UBoS, Uganda Bureau of Statistics; MDAs, Ministries, Departments and Agencies; GBoS, GambiaBureau of Statistics; LGs, local governments; AHSPR, annual health sector performance report; NHP, national health policy; NDP, national development plan; JRM, joint review mission; OCGS, Office of the Commissioner General Statistics.

## Discussion

Tracking the progress being made in countries towards UHC and the SDG 3 necessitates that the planning and implementation of intervention are inextricably linked to M&E.^7^ Against this backdrop, we reviewed the situation analyses of M&E plans for 6 African countries which showed common themes of challenges confronting M&E, some of which were being addressed by the M&E plans whilst others were beyond the scope of the plans. Particularly weak institutional capacity and organisational structures for M&E; fragmentation of M&E functions with inadequate coordination mechanisms; inadequate domestic financing for M&E resulting in donor dependence; limited attention paid to multisectoral approaches, inadequate data availability as well as dissemination and utilization of M&E products for decision-making were common in the situation analyses of the M&E plans of the countries.

These findings corroborate previous analysis and commentaries that also cited inadequate attention paid to the multisectoral collaboration required in data generation and use; weak capacity; coordination inadequacies; the donor-driven and narrow focus health information system as the main drawbacks of M&E in developing countries especially in Africa.^4,18^ Whilst the findings may not be entirely new, their continued existence if not exacerbation despite various efforts in the past signifies their chronicity and potential to undermining the tracking of global and local efforts towards the attainment of UHC and SDG 3. These challenges if not addressed could lead to frustration and resentment towards M&E which would eventually drawback the ability of countries and partners to track the progress (or otherwise) towards the global health agenda. The Kenyan M&E plan characterises this potential situation as health workers at the facility and community level were frustrated by burdensome demands for data while health managers and planners were also frustrated by the competing demands and lack of capacity to adequately respond to those demands; national-level planners becoming frustrated by lack of information relevant to policy and decision-making and; funders (both internal and external) also frustrated because they cannot effectively assess the impact of their support.^25^ The non-use of evidence for decision-making was also cited which perhaps is the conduit for not prioritizing M&E in terms of resource allocation from domestic sources.

Furthermore, the analysis found that the M&E plans of the 6 countries had a total of 614 indicators which varied widely from one country to another, ranging from 38 to 235 indicators. More than half of the total indicators (55.5%) were not classified on a results chain framework. The negative but statistically insignificant relationship between the selection of output indicators and that of outcome indicators observed in this analysis may be underpinned by a seemingly narrow distinction between the outputs and outcome indicators selected by countries. The high number of indicators was found to be attributed to various interests such as funders and programmatic plans, fueled by standalone health information systems.^1,28,29^ Even though there has not been a benchmark of an ‘optimal’ number of indicators for countries in either literature or operational guidelines, having to monitor too many indicators has been shown to be burdensome and could in itself become a drawback to the sustainability of effective M&E especially in the context of countries with suboptimal technological, financial and human resource capacity.^4,30,31^ Therefore, we recommend that as part of the health sector strategic planning process, countries should intensify the M&E planning dialogue to minimise possible redundant indicators, some of which may never get analysed and reported throughout the lifespan of the M&E plan. There is evidence to this effect and an example is the case of Eswatini where at the end term evaluation of the strategic plan, there was no data to report on 60% of the indicators.^32^

Although SDG 3 which is broader in scope than the MDGs embraces efforts to tackle the rising challenge of NCD, the analysis in this review suggests that countries continued to lay greater M&E emphasis on service delivery issues, health systems, maternal and child health as well as communicable diseases as was seen in the MDG era. There is a seeming neglect of the NCDs and their risk factors which are fast becoming the leading cause of morbidity and mortality in many countries and accounted for 71% of global deaths in 2016.^32^ If the trend of not adequately prioritizing NCDs indicators in M&E plans is widespread beyond the sampled country M&E plans under review, it could have dire consequences for tracking the progress made in the fight against NCDs including injuries which will ultimately undermine the first of triple billion targets of WHO which is “1 billion more people benefiting from UHC” and SDG 3 in general. Therefore, beyond providing M&E frameworks and compendium of indicators to guide countries, we advocate for a more binding minimum set of indicators which could be added upon by countries depending on their context and needs.

Added to the aforesaid, the analysis revealed that despite the numerous indicators selected in the country M&E plans, they averagely included only 58% (range: 48%-67%) of the SDG 3 indicators. Indeed, countries that had the most indicators tended to include fewer SDG 3 monitoring indicators. This implies that in the countries under consideration, the routine monitoring mechanism may not provide sufficient and relevant information for comprehensive monitoring of the SDG 3 targets. Thus, they may end up needing additional effort to be able to fulfil the SDG 3 monitoring requirement which may, in turn, reinforce the challenges of non-integration and multiplicity of reporting requirements against a backdrop of limited funding. Furthermore, there was suboptimal consideration for equity analysis in the reviewed plans. The resource implications of extensive equity analysis on a routine basis could justify some countries adopting an incremental approach by starting with a minimal stratification with the hope of adding more as the M&E systems become more robust.

There are global efforts to support strengthening M&E at country level and among these is the Health data collaborative which seeks to align partner resources and interventions to the country M&E, engage a wider set of actors including the private sector and tracking progress in country capacity to monitor the health-related SDGs. There are positive lessons from Kenya with regards to partner alignment to one M&E plan.^19^

### Strengths and Limitations of the Study

The health sector’s vision in strengthening M&E is laid down in M&E plans which guides investments and interventions of all actors. Our assessment of M&E plans gives inference on the likely robustness of M&E systems to monitor SDG 3 targets. We however note that a good plan may not necessarily translate into strong M&E systems because the implementation step is not always guaranteed. We reviewed few M&E plans,^6^ the findings from these as well as the recommendations made, may not be representative of the African region. We acknowledge that some of the SDG 3 indicators may be monitored through other M&E plans and as such missed in our review. In addition, we reviewed publicly available plans, additional challenges and insight may have been gained through interviews with key stakeholders in these countries. We however believe there are important lessons that can inform improvement in planning for M&E for SDG 3. Three of the plans (Kenya, The Gambia, and Zanzibar) were developed prior to the starting date of implementing SDGs, however, these were still guiding M&E activities and as such we justified to review them.

## Conclusion

With more ambitious international health goals and targets together with increasing demand for social accountability in health, there is a growing interest in assessing the performance of health systems especially in developing countries where health system efficiencies continue to raise concerns.^4,33^ An analysis of M&E plans of 6 countries in the WHO Africa Region re-echoed common themes of challenges confronting effective M&E at county level such as weak institutional capacity; weak coordination; inadequate domestic funding for M&E and suboptimal data availability. Amidst the global affirmation of the SDG 3 and UHC by all countries, the analysis showed that inadvertently or otherwise, countries had omissions of indicators for monitoring key components of the SDG 3 targets especially those on NCDs and injuries in favour of targets that were the bedrock of the MDG era. We conclude that on the back of the numerous challenges confronting effective M&E at county level, and the high number of indicators in the M&E plans majority of which are not properly aligned to logical results chains and the SDG 3 indicators, these could prove to be significant bottlenecks to tracking progress if they are not addressed. Therefore, increased efforts in strengthening M&E capacity in countries is strongly advocated for while MoHs should also prioritise M&E as an important pillar for health service planning and implementation; not an ad-hoc add-on activity. Additionally, a critical assessment of the relevance of an indicator needs to be undertaken to ensure that a manageable number of indicators are selected that can adequately assess health sector performance and progress towards UHC. We further contend that the 27 indicators monitoring the 13 targets of SDG 3 should be given serious consideration by countries if not regarded as the minimum indicator set when developing M&E plans.

## Ethical issues

We reviewed M&E plans that were in the public domain. As such, ethical approval was not required.

## Competing interests

Authors declare that they have no competing interests.

## Authors’ contributions

JNO conceptualized the study and led the drafting of the manuscript, NJB, AJA, and JNO undertook review of the plans and data analysis. NJB and AJA participated in the data analysis and drafting of the manuscript. All authors reviewed and approved the final manuscript.

## Authors’ affiliations

^1^HealthNet Consult, Kampala, Uganda. ^2^World Health Organization (WHO), Inter-Country Support Team for Eastern & Southern Africa, Harare, Zimbabwe.

## Key messages

**Implications for policy makers**

- The Millennium Development Goals (MDGs) era availed opportunities for scaling up service coverage but also called for stringent monitoring and evaluation (M&E) requirements focussing mainly on MDG related programs (maternal and child health, HIV, tuberculosis and malaria). The Sustainable Development Goals 3 (SDG 3) ensure healthy lives and promote well-being for all at all ages; and the universal health coverage (UHC) agenda present a broader scope and call for more sophisticated M&E systems.
- Previous efforts in strengthening M&E have been characterised by vertical approaches, fragmentation and underfunding resulting in suboptimal performance of the M&E systems.
- The country adaptation of monitoring for SDG 3 and UHC as reflected in M&E plans shows varied considerations for the major parameters that must be reflected to adequately monitor UHC.
- The twenty-seven indicators for monitoring the 13 targets of SDG 3 should be given serious consideration by countries if not regarded as the minimum indicator set when developing M&E plans.

**Implications for the public**

Attaining Sustainable Development Goal 3 (SDG 3) targets and more important reaching universal health coverage (UHC) is every county’s aspiration but the starting point, as well as the path will be unique to each country. This underscores the need for strong monitoring and evaluation (M&E) to assess progress and in this regard, availability of M&E plans is crucial. Of concern however, are the institutional and systemic challenges to M&E; the burden of a high number of indicators selected; omission of some SDG 3 targets specifically for injuries, non-communicable diseases (NCDs) and associated risk factors and; the limited consideration for the much desired multisectoral collaboration in M&E for SDG 3 and UHC. Attaining UHC is a journey and M&E is key to ensuring progress. Our assessment provides important transferable lessons that can be contextualised to inform improvement in planning for M&E for SDG 3.
